# Therapeutic Potential of the Combination of Pentoxifylline and Vitamin-E in Inflammatory Bowel Disease: Inhibition of Intestinal Fibrosis

**DOI:** 10.3390/jcm11164713

**Published:** 2022-08-12

**Authors:** Hyun Joo Lee

**Affiliations:** 1 Division of Gastroenterology, Department of Internal Medicine, CHA Bundang Medical Center, CHA University, 59 Yatap-ro, Bundang-gu, Seongnam 13496, Korea; mm888@naver.com; Tel.: +82-31-881-7075; 2 Division of Gastroenterology, Department of Internal Medicine, Graduate School, CHA University, 335 Pangyo-ro, Bundang-gu, Seongnam 13488, Korea

**Keywords:** inflammatory bowel disease, intestinal fibrosis, pentoxifylline, vitamin-E, myofibroblasts, extracellular matrix

## Abstract

Background: Although intestinal fibrosis is a consequence of recurrent inflammation in Inflammatory bowel disease (IBD), alleviating inflammation alone does not prevent the progression of fibrosis, suggesting that the development of direct anti-fibrotic agents is necessary. This study aimed to evaluate the anti-fibrotic properties of combination treatment with pentoxifylline (PTX) and vitamin E (Vit-E) on human primary intestinal myofibroblasts (HIMFs) and the therapeutic potential of the combination therapy in murine models of IBD. Methods: HIMFs were pretreated with PTX, Vit-E, or both, and incubated with TGF-β1. We performed Western blot, qPCR, collagen staining, and immunofluorescence to estimate the anti-fibrotic effects of PTX and Vit-E. The cytotoxicity of these was investigated through MTT assay. To induce murine models of IBD for in vivo study, C57BL/6 mice were treated with repeated cycles of dextran sulfate sodium (DSS), developing chronic colitis. We examined whether the combined PTX and Vit-E treatment would effectively ameliorate colonic fibrosis in vivo. Results: We found that the co-treatment with PTX and Vit-E suppressed TGF-β1-induced expression of fibrogenic markers, with decreased expression of pERK, pSmad2, and pJNK, more than either treatment alone in HIMFs. Neither PTX nor Vit-E showed any significant cytotoxicity in given concentrations. Consistently with the in vitro results, the co-administration with PTX and Vit-E effectively attenuated colonic fibrosis with recovery from thickening and shortening of colon in murine models of IBD. Conclusions: These findings demonstrated that the combination of PTX and Vit-E exhibits significant anti-fibrotic effects in both HIMFs and in vivo IBD models, providing a promising therapy for IBD.

## 1. Introduction

Inflammatory bowel disease (IBD), including Crohn’s disease (CD) and ulcerative colitis (UC), is manifested by chronic inflammation of the gastrointestinal tract with significant morbidity and sometimes life-threatening complications [1,2]. Intestinal fibrosis is a serious and common complication of IBD, which is clinically more definite in CD [2,3,4,5]. Up to one third of CD patients will eventually suffer from end-stage fibrotic disease followed by intestinal strictures, resulting in obstruction and necessitating surgery [6,7,8,9]. While current anti-inflammatory therapies may relieve inflammatory strictures, fibrostenotic strictures are not resolved by anti-inflammatory agents such as immunosuppressants [10]. Although intestinal fibrosis is a consequence of recurrent inflammation, alleviating inflammation alone does not prevent or reverse the progression of fibrosis, suggesting that the development of direct anti-fibrotic agents is necessary [11,12]. There have been several studies regarding therapeutic targets (TGF-β pathways, TIMP/MMP balance, VEGF, FAP, EMT, IL-17, IL-36, NETs, intestinal microbiota etc.) and the relevant target-specific molecules for intestinal fibrosis. However, to date, no anti-fibrotic agents are clinically available to prevent or reverse intestinal fibrosis [13].

The cytokine transforming growth factor-β1 (TGF-β1) is a key mediator of tissue repair and healing and also has important implications in progressive tissue fibrosis [14,15,16]. It is also a pivotal profibrotic cytokine, involved in the pathogenesis of intestinal fibrosis in IBD [4,17,18,19,20]. The fibrogenic activation of intestinal myofibroblasts is modulated by Smad-dependent and Smad-independent TGF-β signaling pathways [21,22]. Smad-dependent TGF-β signaling causes the phosphorylation of Smad2 and Smad3, which combine with Smad4, and the Smad complex translocates to the nucleus [1,22,23,24,25]. The Smad3 component of the complex directly binds to gene promoters to induce the transcription of profibrotic molecules, including α-smooth muscle actin (α-SMA), collagens, fibronectin and connective tissue growth factor (CTGF) [26]. Smad-independent TGF-β signaling promotes the phosphorylation of extracellular signal-regulated kinase (ERK), c-Jun N-terminal kinase (JNK), p38 mitogen-activated protein kinase (MAPK), Akt, and myosin light chain (Rho signaling) [23,27,28,29]. Targeting the TGF-β1 signaling pathways is key to the development of a novel anti-fibrotic agent for IBD patients. Indeed, some molecules, including captopril, AMA0825, cilengitide, and pirfenidone, have been shown to have inhibitory effects on intestinal fibrosis in vitro or in vivo by downregulating the TGF-β1 signaling [13,21,30,31,32,33,34].

With efforts being made to develop a new therapeutic agent for IBD, previous findings showed that pentoxifylline (PTX) and its metabolite-1 exerted beneficial effects on colonic inflammation and fibrosis in 2,4,6-trinitrobenzene sulfonic acid (TNBS)-colitis models [35,36]. PTX is a well-known methylxanthine derivative and non-selective phosphodiesterase (PDE) inhibitor, which has potent anti-fibrotic activity, and has been proven to prevent liver fibrosis, radiation-induced fibrosis, and renal fibrosis [37,38,39,40,41]. In 1984, FDA approved its use for intermittent claudication in the United States, and it has been used worldwide for the treatment of intermittent claudication [42]. However, according to the study of Berman B et al., PTX decreased the synthetic capacity of fibroblasts by inhibiting the production of collagen, fibronectin, and glycosaminoglycan (GAG) and promoting collagenase production. Moreover, PTX suppressed the proliferation of fibroblasts without lethal toxicity [43].

Vitamin-E (Vit-E, α-tocopherol) is an important antioxidant that scavenges free radicals, and thus protects cells against damage and the human body against aging. Vit-E has also been reported to diminish the expression of genes involved in fibrotic process, such as matrix metalloproteinase-1 (MMP-1), IL-1β, or collagen [44,45,46], and delay the progression of liver fibrosis, radiation-induced fibrosis, and renal fibrosis [47,48,49,50,51]. Its anti-fibrotic effect is known to be mainly mediated by the inhibition of TGF-β1 signaling pathways, but the underlying mechanism needs to be further explored.

Combination therapy with PTX and Vit-E has been reported to exhibit potent anti-fibrotic effect in previous studies [39,52,53,54]. Those anti-fibrotic effects are known to be primarily related to the blockade of TGF-β signaling pathways [39,50,55,56,57]. However, to date, combination therapy with PTX and Vit-E has not been investigated in a model of intestinal fibrosis in IBD. Therefore, this study aimed to evaluate the anti-fibrotic properties of combination treatment with PTX and Vit-E on human primary intestinal myofibroblasts (HIMFs) and the therapeutic potential of the combination therapy in murine models of IBD. Furthermore, we could unravel the mechanisms by which the combination therapy impedes intestinal fibrosis in IBD.

## 2. Materials and Methods

### 2.1. Isolation of Human Primary Intestinal Myofibroblasts and Cell Culture

HIMFs were isolated from normal colon segments of patients undergoing resection surgery for colorectal cancer. The grossly normal colon segments were obtained around the proximal resection margin by a pathologist after surgical resection. Their periphery was histologically confirmed through a microscope. The project was implemented in accordance with the guidelines of the Institutional Review Board of the CHA Bundang Medical Center. HIMFs were cultured in high-glucose Dulbecco’s modified Eagle’s medium (DMEM), supplemented with 10% FBS, 4 mmol/L L-glutamine, 25 mmol/L HEPES, 100 U/mL penicillin, 100 μg/mL streptomycin, and 0.25 μg/mL amphotericin B and incubated at 37 °C in air containing 5% CO_2_.

### 2.2. Pharmacologic Treatment

PTX powder (Pentoxifylline, M.W. 278.31, Sigma-Aldrich, St. Louis, MO, USA) was solubilized in autoclaved water and 100 mM solution was made. The stock solution of Vit-E (α-tocopherol, M.W. 430.71, Sigma-Aldrich) was solubilized in dimethyl sulfoxide (DMSO) and 100 mM solution was prepared. Finally, 100 µM–2 mM solution of PTX or Vit-E was produced from dilutions of the 100 mM solution by using serum free media. Recombinant human TGF-β1 was purchased from R&D systems (Minneapolis, MN, USA). For cytotoxicity assay, HIMFs were incubated under the treatment with various concentrations of PTX or Vit-E. To examine anti-fibrotic activities of PTX and Vit-E, HIMFs were pretreated with PTX, Vit-E, or both for 30 min and then incubated with 5 ng/mL of TGF-β1 for 24 h or 48 h.

Performing animal experiments, while PTX was dissolved in phosphate buffered saline (PBS), Vit-E was dissolved in PBS:ethanol =1:1 solvent. Then, 50 mg/kg PTX with or without Vit-E was intraperitoneally (i.p.) injected to dextran sulfate sodium (DSS)-induced IBD models. Vehicle was injected i.p. to the DSS control group.

### 2.3. Cytotoxicity Assay

The cytotoxicity of PTX and Vit-E was investigated through MTT assay (MTT assay kit, ab211091, Abcam, Cambridge, UK). First, HIMFs were seeded into 96-well pates at a density of 1 × 10^4^ cells per well and incubated for 24 h/48 h in the presence of various concentrations (100 µM-2 mM) of PTX or Vit-E. Later, a series of MTT assay procedures were conducted, and finally, cell viability was measured from a microplate reader at absorbance 590 nm.

### 2.4. Quantitative Real-Time PCR

HIMFs were pretreated with/without PTX, Vit-E, or both (1 mM) for 30 min and then TGF-β1 (5 ng/mL) was added to the media and the cultures were incubated for a further 24 h. Total cellular RNA was extracted using TRIzol reagent (Ambion, Carlsbad, CA, USA), quantified by a NanoDrop spectrophotometer, and complementary DNA was synthesized as per TOYOBO ReverTra^®^ Ace qPCR RT Master Mix Kit (TOYOBO, Osaka, Japan). Real-time PCR analysis of the mRNA expression of *COL1A1*, *ACTA2*, and *FN1* was implemented using cDNA products, gene-specific primers, and Faster-Start Essential DNA Probes Master (Roche) on a Roche Light Cycler 96 instrument. The specific primers for *COL1A1* (Hs00164004_m1), *ACTA2* (Hs00426835_g1), *FN1* (Hs01549976_m1), and *GAPDH* (Hs03929097_g1) genes were purchased from Applied Biosystems (Foster City, CA, USA). Expression levels of the target mRNAs were normalized to *GAPDH*.

### 2.5. Western Blot Analysis

HIMFs were pretreated with/without PTX, Vit-E, or both (1 mM, 2 mM) for 30 min and then TGF-β1 (5 ng/mL) was added to the media and the cultures were incubated for a further 48 h. Protein lysates of HIMFs were isolated as per protein extraction protocol and quantified by BCA assay (Pierce^®^ BCA Protein assay, #23228, Thermo Fisher Scientific, Waltham, MA, USA). A total of 20–30μg of protein from each sample was prepared, separated by electrophoresis on 10% polyacrylamide gels, transferred to PVDF membranes, and probed with the following primary Abs at the indicated dilutions; Procol1A1, 1:20 (SP1D8, Developmental Studies Hybridoma Bank, Iowa City, IA, USA); α-SMA, 1:5000 (A2547, MilliporeSigma, Burlington, MA, USA); CTGF, 1:1000 (sc-101586, Santa Cruz Biotechnology, Santa Cruz, CA, USA); FN, 1:10,000 (ab2413, Abcam); pERK1/2 Thr202/Tyr204, 1:1000 (#9101, Cell Signaling Technology, Danvers, MA, USA); ERK1/2, 1:1000 (#9102, Cell Signaling Technology); pSmad2 Ser465/467, 1:1000 (#3108, Cell Signaling Technology); Smad2, 1:1000 (#3122, Cell Signaling Technology); pJNK Thr183/Tyr185 1:1000 (#4668, Cell Signaling Technology); GAPDH, 1:5000 (#2118, Cell Signaling Technology). Subsequently, the membranes were incubated with diluted horseradish peroxidase-conjugated anti-rabbit or anti-mouse secondary Abs (GeneTex, Irvine, CA, USA). The protein bands were visualized using the SuperSignal West Pico Chemiluminescence System (Thermo Fisher Scientific). Quantification of the interested proteins was performed by Image J program.

### 2.6. Sirius Red/Fast Green Collagen Staining

Sirius red/Fast green Collagen staining (#9046, Chondrex Inc., Redmond, WA, USA) was employed to measure the amount of intracellular collagen. In brief, after being seeded into 24-well plates at a density of 3 × 10^4^ cells per well and stimulated by TGF-β1 (5 ng/mL) for 24 h with the 30 min-pretreatment of PTX/Vit-E (1 mM), HIMFs were fixed in Kahle fixative, washed with PBS, stained with dye solution, washed with distilled water, and gently mixed with dye extraction buffer until the color was eluted from each sample. Finally, the amount of intracellular collagen was measured with a microplate reader at absorbance 540/605 nm.

### 2.7. Immunofluorescence Microscopy

HIMFs were seeded into chamber slides (#30108, SPL life Sciences, Pocheon-si, Korea) at a density of 1 × 10^4^ cells per well, pretreated with/without PTX, Vit-E, or both (1 mM) for 30 min; then, TGF-β1 (5 ng/mL) was added to the media, and the cultures were incubated for a further 48 h. The cells were fixed in 4% paraformaldehyde, permeabilized in 0.1% Triton X-100, blocked in 5% bovine serum albumin (BSA), and then labeled with the following primary Abs at the indicated dilutions; Procol1A1, 1:20; α-SMA, 1:500; FN, 1:500. These Abs were described in the Western blot. After washing with PBS, the cells were treated with AlexaFluor488/594-conjugated goat anti-mouse/rabbit secondary Ab (Molecular Probes, Eugene, OR, USA) for 2 h. After washing, the slides were mounted with mounting solution containing DAPI (Abcam, ab104139) and photographed in a Zeiss LSM880 confocal laser scanning microscope.

### 2.8. DSS-Induced IBD Murine Models

Six-week-old female C57BL/6 mice were purchased from Orient Bio Inc., Seongnam-si, Korea. After one-week-adaptation, chronic colitis was induced by oral administration of dextran sulfate sodium (DSS, M.W. 36–50 kDa, MP Biomedicals, CA, USA). 1.5–2.5% DSS was added to drinking water. Mice were treated with 3 cycles of DSS for 5 days and 5 days of drinking water between each cycle. Mice were divided into 4 experimental groups (n = 5 per group) and DSS-induced colitis mice were randomized to 3 groups: (A) Normal; (B) DSS + vehicle; (C) DSS + PTX 50 mg/kg; (D) DSS + PTX/Vit-E 50 mg/kg. PTX and Vit-E were given i.p. at 50 mg/kg from day 16 to day 35. Body weight, stool consistency, and presence of hematochezia were monitored daily for 35 days. After 35 days, mice were sacrificed, and tissue samples were harvested. All animal care and experimental procedures in this study were done in accordance with the guidelines for animal experiments in the University of CHA with the approval of the Institutional Animal Care and Use Committee (IACUC, IACUC210131). All efforts, including euthanasia, were made to reduce suffering.

### 2.9. Clinical Disease Scoring

During the experimental period, the severity of colitis was assessed daily by recording the percentage of body weight change and disease activity index (DAI). The DAI score was calculated as the sum of the score for relative body weight loss, stool consistency, and presence of hematochezia. Body weight loss was scored as follows; score 0, no weight loss compared to initial weight; 1, weight loss within 0–10%; 2, weight loss within 11–15%; 3, weight loss within 16–20%; 4, weight loss greater than 20%. Stool consistency was determined as follows; score 0, normal (solid pellet); 2, loose stool; 4, diarrhea. Rectal bleeding score was graded as follows; score 0, absence; 4, gross bleeding.

### 2.10. Macroscopic Assessment and Histological Analysis

On day 35, the mice were euthanized using CO_2_ gas, and the length and weight of those colons were measured. The colon length was measured from the cecum to the anus. The fresh colon tissues were washed with cold PBS, cut transversely into several parts, one or two of which were fixed in 4% paraformaldehyde, and embedded in paraffin. Paraffin-embedded colon sections were routinely prepared and stained with hematoxylin and eosin (H&E). Colon wall thickness was determined in the sections from eight random places in each representative area.

### 2.11. Quantification of Fibrosis

To evaluate the ability of the combination of PTX and Vit-E and effectively attenuate intestinal fibrosis in animal models of IBD, colon tissue sections were stained with Sirius red or Masson’s trichrome according to standard methods. The red area of Sirius red staining and the blue area of trichrome staining were considered fibrotic, and quantification of tissue fibrosis was carried out by image J program.

### 2.12. Statistical Analysis

All data were expressed as mean ± SEM and statistically analyzed using one-way ANOVA followed by Dunnett’s test (among multiple groups) or unpaired two-tailed Student’s *t*-test (for comparison between the 2 groups). The differences were considered significant if *p* < 0.05. Statistical analyses were carried out with GraphPad Prism software v9.3.1 (GraphPad Software Inc., San Diego, CA, USA).

## 3. Results

### 3.1. Given Concentrations of PTX or Vit-E Have No Significant Cytotoxicity against HIMFs

**Figure 1:**
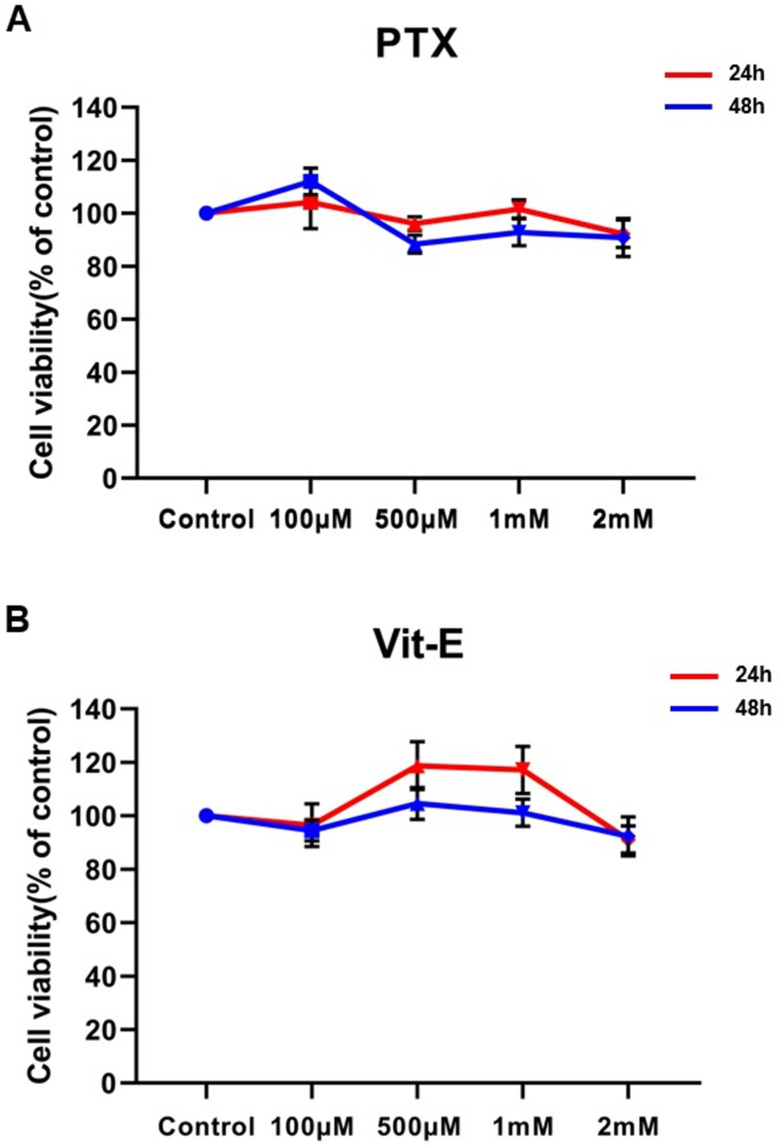
Neither PTX nor Vit-E shows any detectable cytotoxic effect against HIMFs at concentrations ranging from 0.1 mM to 2 mM. (**A**,**B**) HIMFs were incubated with various concentrations (0.1–2 mM) of PTX or Vit-E for 24/48 h, after which cell viability was assessed by MTT assay (n = 4). The optical density value of the control was regarded as 100%. Cell viability was calculated as a percentage of the control and expressed as mean±SEM. The data were analyzed by one-way ANOVA, followed by Dunnett’s test. *p* < 0.05 was considered significant.

We investigated the cytotoxicity of various concentrations of PTX and Vit-E through MTT assay. HIMFs were incubated under treatment with 0.1–2 mM of PTX or Vit-E for 24 h/48 h, and cell viability was measured. Exposure to 0.1–2 mM of PTX or Vit-E did not show any significant cytotoxic effect against HIMFs, indicating that any concentration ranging from 0.1 mM to 2 mM is suitable for evaluating the anti-fibrotic properties of PTX and Vit-E in HIMFs (Figure 1).

### 3.2. The Co-Treatment with PTX and Vit-E Suppresses TGF-β1-Induced Expression of Fibrogenic Markers More than Either Treatment Alone in HIMFs

**Figure 2:**
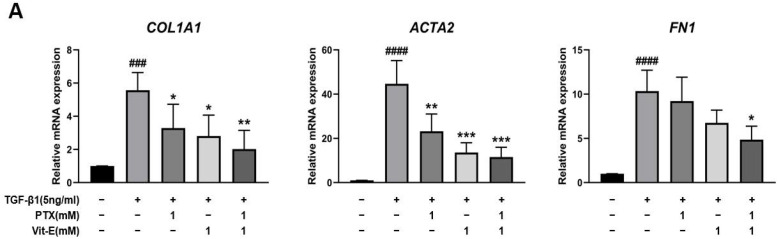

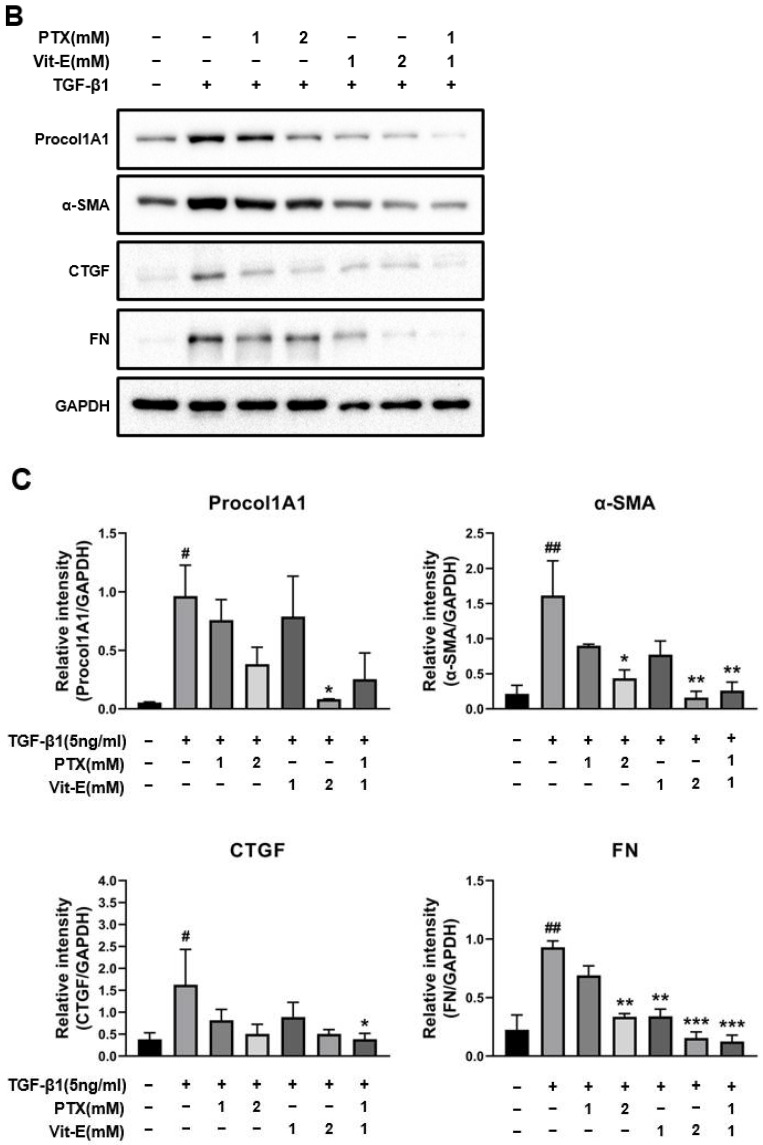

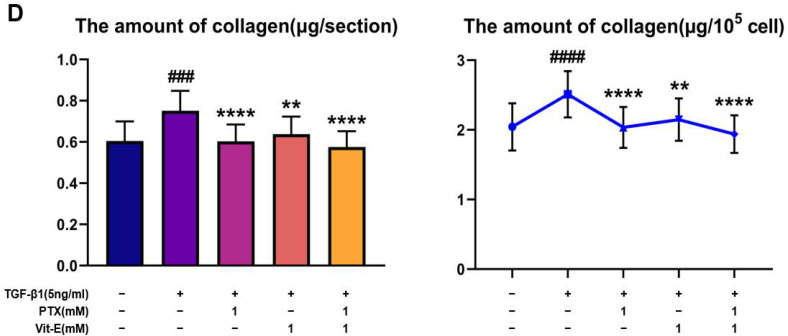
Each PTX and Vit-E exhibits anti-fibrotic activities and the combination of these two agents produces enhanced beneficial effects in TGF-β1-stimulated HIMFs. (**A**–**C**) To determine the roles of PTX and Vit-E on the expression of fibrotic markers, HIMFs were pretreated with/without PTX, Vit-E, or both (1 mM, 2 mM) for 30 min, and then TGF-β1 (5 ng/mL) was added to the media and the cultures were incubated for a further 24 h (in qPCR) or 48 h (in Western blot). The expression of ECM components and α-SMA was investigated in protein/mRNA levels for Western blot and qPCR analysis. (**A**) The relative mRNA expression levels of *COL1A1*, *ACTA2*, and *FN1* with *GAPDH* as the endogenous control. PTX and Vit-E (1 mM) each inhibited the mRNA expression of the fibrotic markers, which was enhanced by TGF-β1. Combined PTX and Vit-E treatment (1 mM) demonstrated a greater anti-fibrotic effect than either treatment alone. (**B**,**C**) Representative Western blots for the protein expression levels of Procol1A1, α-SMA, CTGF, and FN, with GAPDH as the endogenous control. Individual treatment with PTX or Vit-E (1 mM, 2 mM) tended to dose-dependently suppress TGF-β1-stimulated expression of the fibrogenic proteins and the combination of these two agents (1 mM) was also proven to reduce the expression of fibrogenic molecules more than either treatment alone (1 mM). (**D**) After co-incubation with/without PTX, Vit-E, or both (1 mM) on the stimulation by TGF-β1 (5 ng/mL), the amount of intracellular collagen was estimated via Sirius Red/Fast Green Collagen staining (n = 5). The amount of collagen per section or 10^5^ cell was shown on graphs. These results suggest that TGF-β1-induced collagen accumulation was mitigated with PTX and Vit-E pretreatment. (**A**–**D**). The data were expressed as mean ± SEM and analyzed by one-way ANOVA, followed by Dunnett’s test. *p* < 0.05 was considered significant (^#^ *p* < 0.05; ^##^ *p* < 0.01; ^###^ *p* < 0.001; ^####^ *p* < 0.0001 versus the control; * *p* < 0.05; ** *p* < 0.01; *** *p* < 0.001; **** *p* < 0.0001 versus the TGF-β1-treatment-alone).

**Figure 3:**
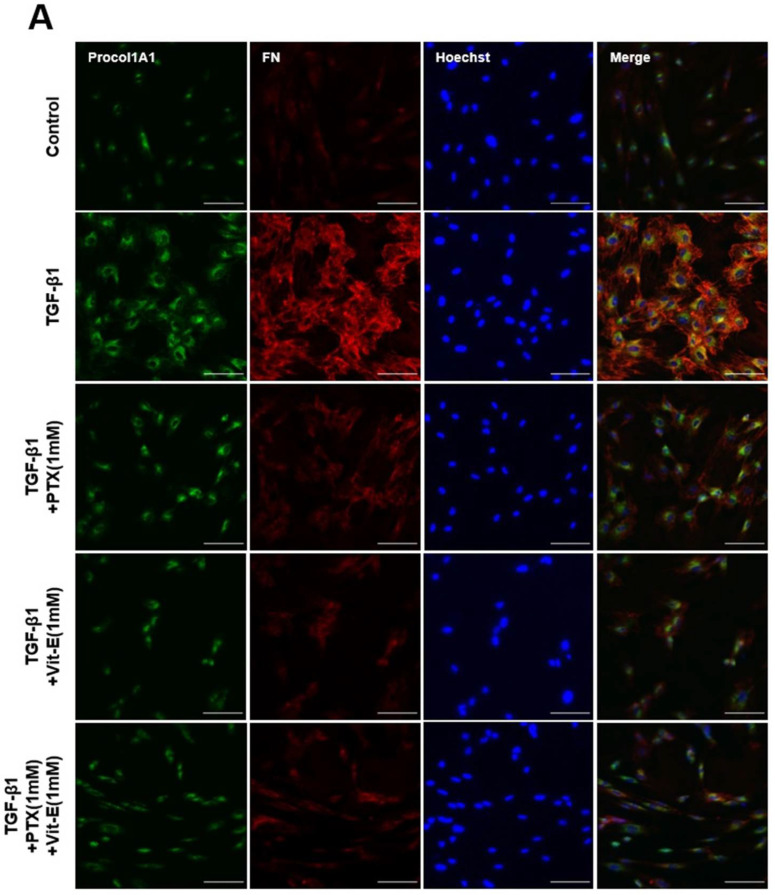

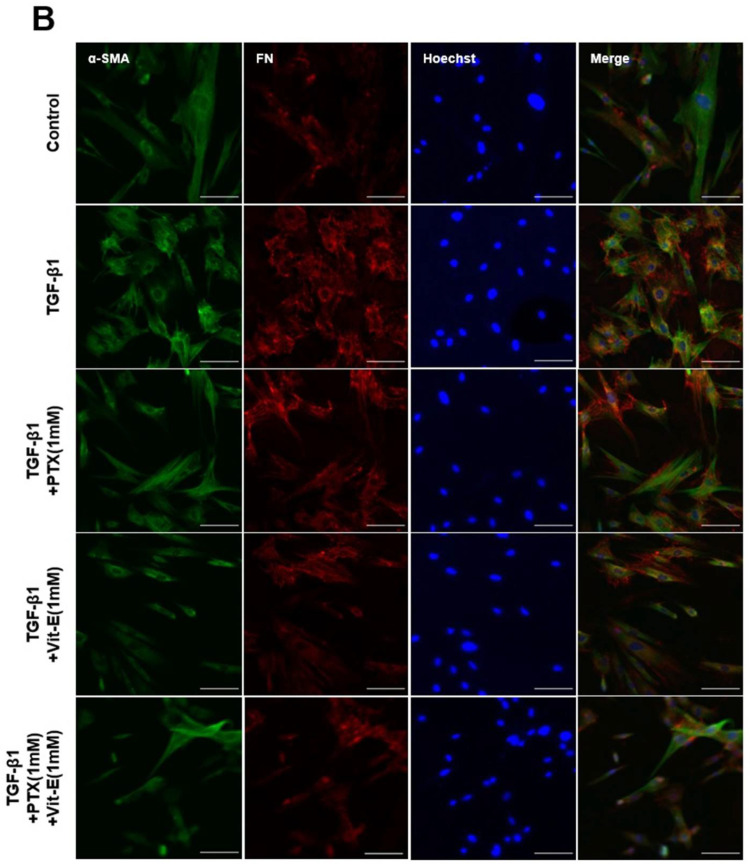
Immunofluorescence staining represents that PTX and Vit-E attenuate TGF-β1-stimulated expression of fibrotic markers on HIMFs. (**A**,**B**) Confocal microscopic images of the expression of Procol1A1, FN, and α-SMA on HIMFs to identify the effects of TGF-β1, PTX, and Vit-E. HIMFs were pretreated with/without PTX, Vit-E, or both (1 mM) for 30 min and then TGF-β1 (5 ng/mL) was added to the media and the cultures were incubated for a further 48 h. After incubation, HIMFs were immunostained with Procol1A1, FN, and α-SMA Abs and counterstained with Hoechst. α-SMA immunostaining displayed well-organized, intensely stained actin stress fibers and immunostaining of Procol1A1 and FN also showed strong signals in TGF-β1-stimulated HIMFs. The PTX/Vit-E treatment prominently diminished the signal intensities of those. HIMFs were shown as follows: Procol1A1, green; FN, red; α-SMA, green; DAPI for nucleus staining, blue. Original magnification × 200, scale bar 100 µm.

To estimate the anti-fibrotic activities of PTX and Vit-E, HIMFs were incubated with TGF-β1 (5 ng/mL) after pretreatment with PTX, Vit-E, or both (1 mM, 2 mM), and we assessed the expression of extracellular matrix (ECM) components (e.g., Col1A1, FN, CTGF) and α-SMA on HIMFs in protein/gene levels using Western blot and qPCR analysis. As shown in Figure 2A, PTX and Vit-E (1 mM) each inhibited the mRNA expression of *Col1A1*, *ACTA2*, and *FN1*, which was enhanced by TGF-β1. Combined PTX and Vit-E treatment (1 mM) substantially subdued mRNA expression of the fibrosis-related molecules more than single treatments. Likewise, in Western blot, we found that each of these (1 mM, 2 mM) was inclined to suppress TGF-β1-stimulated expression of the fibrogenic proteins, including α-SMA, Procol1A1, CTGF, and FN, in a dose-dependent manner, and the combination of these (1 mM) suppressed the upregulated expression of the ECM components and α-SMA in response to TGF-β1 to a greater extent than individual treatments (Figure 2B,C). For Sirius Red/Fast Green Collagen staining, TGF-β1-promoted collagen production was proven to be mitigated by pretreatment with PTX and Vit-E (1 mM) in HIMFs (Figure 2D). Data were displayed on graphs, presented as mean ± SEM by the μg per section or the μg per 10^5^ cell. We further substantiated the potent anti-fibrotic effects of PTX and Vit-E via immunofluorescence. As represented in Figure 3, α-SMA immunostaining displayed well-organized, intensely stained actin stress fibers, and immunostaining of Procol1A1 and FN also showed strong signals in TGF-β1-stimulated HIMFs. The PTX and Vit-E treatment (1 mM) prominently abated the signal intensities of those.

### 3.3. The Combination of PTX and Vit-E Exhibits Anti-Fibrotic Efects through Inhibition of TGF-β1-Mediated Downstream Signaling, including Both Smad-Dependent and Smad-Independent Pathways

**Figure 4:**
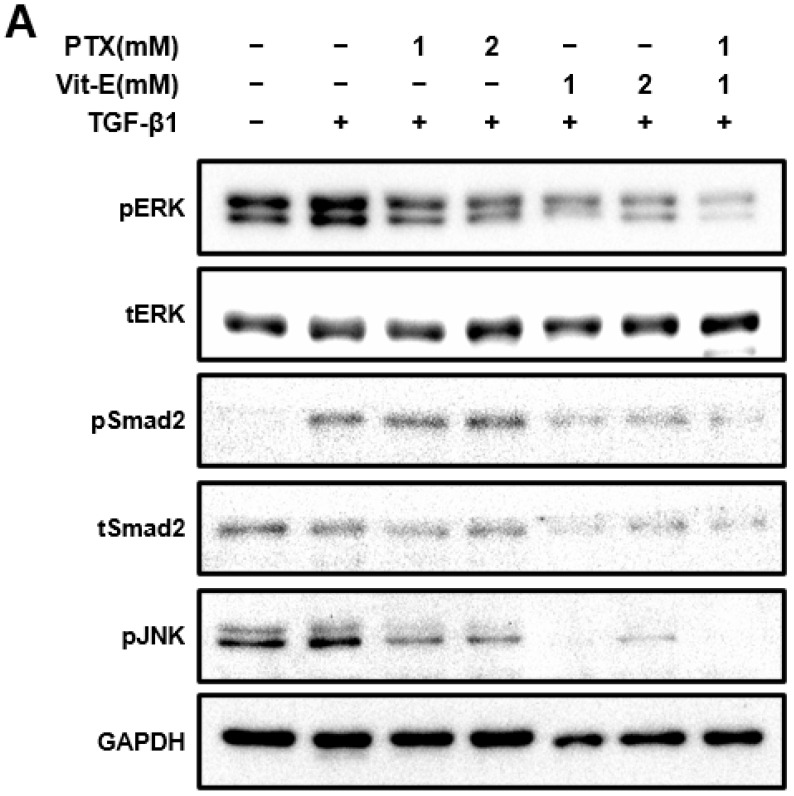

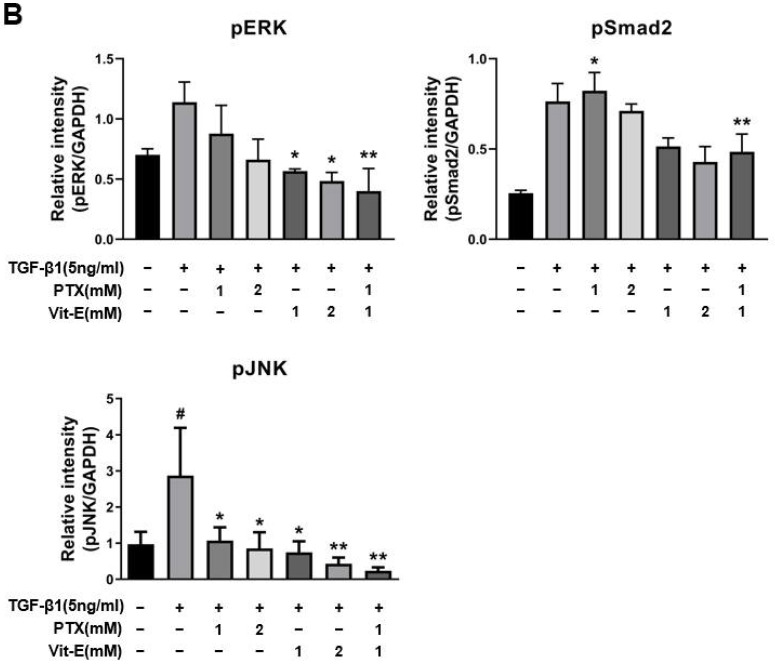
The combination of PTX and Vit-E has potent anti-fibrotic effects via the disruption of TGF-β1-mediated downstream signaling. (**A**,**B**) Representative Western blots for the protein expression of pERK, pSmad2, and pJNK with GAPDH as the endogenous control. pERK, pSmad2, and pJNK were defined as phosphorylated ERK, phosphorylated Smad2, and phosphorylated JNK, respectively. Total ERK and Smad2 proteins were also analyzed and expressed as tERK and tSmad2, respectively. As is well-known from prior studies, TGF-β1 reinforced phosphorylation of downstream targets involved in fibrogenesis in HIMFs. When HIMFs were pretreated with PTX, Vit-E, or both (1 mM, 2 mM) for 30 min and then TGF-β1 (5 ng/mL) was added to the media and the cultures were incubated for a further 48 h, phosphorylation of ERK was dose-dependently inhibited and downregulated the most in the combined PTX and Vit-E treatment (1 mM). Likewise, PTX and Vit-E decreased JNK phosphorylation and the combination treatment (1 mM) suppressed the most the phosphorylation. It was interesting that the Vit-E pretreatment inhibited Smad2 phosphorylation, whereas the PTX pretreatment did not significantly affect the phosphorylation. The combination of PTX and Vit-E had the same effect on Smad2 phosphorylation as Vit-E alone. (**B**) The relative intensities of the protein expression of pERK, pSmad2, and pJNK with GAPDH as the endogenous control. The data were expressed as mean ± SEM and analyzed by one-way ANOVA, followed by Dunnett’s test. *p* < 0.05 was considered significant (^#^ *p* < 0.05 versus the control; * *p* < 0.05; ** *p* < 0.01 versus the TGF-β1-treatment-alone).

To explore the mechanisms by which PTX and Vit-E exert anti-fibrotic effects and how the combination of these exerts a greater anti-fibrotic effect on HIMFs, we used Western blot analysis to examine whether individual treatment or co-treatment is correlated with the RAS/MEK/ERK, JNK, or Smad signaling pathways (Figure 4). Phosphorylation of ERK was dose-dependently inhibited in a single treatment of PTX or Vit-E (1 mM, 2 mM) and downregulated the most in the combination treatment of PTX and Vit-E (1 mM), compared with the TGF-β1-treatment-alone (*p* < 0.01). Likewise, the individual treatment of PTX or Vit-E (1 mM, 2 mM) decreased JNK phosphorylation and the combination treatment (1 mM) decreased this the most (*p* < 0.01). It was interesting that the Vit-E pretreatment inhibited Smad2 phosphorylation, whereas PTX pretreatment did not significantly affect the phosphorylation. The combination of PTX and Vit-E had the same effect on Smad2 phosphorylation as Vit-E alone, indicating that PTX in the presence of Vit-E did not affect Smad2 phosphorylation. Consequently, the underlying mechanism by which either PTX or Vit-E exhibits anti-fibrotic activities and the combination of those elicits a greater anti-fibrotic effect on HIMFs must be linked to the suppression of TGF-β1-mediated downstream signaling, including both Smad-dependent and Smad-independent pathways.

### 3.4. The Combination of PTX and Vit-E Improves In Vivo Clinical Indicators of IBD

**Figure 5:**
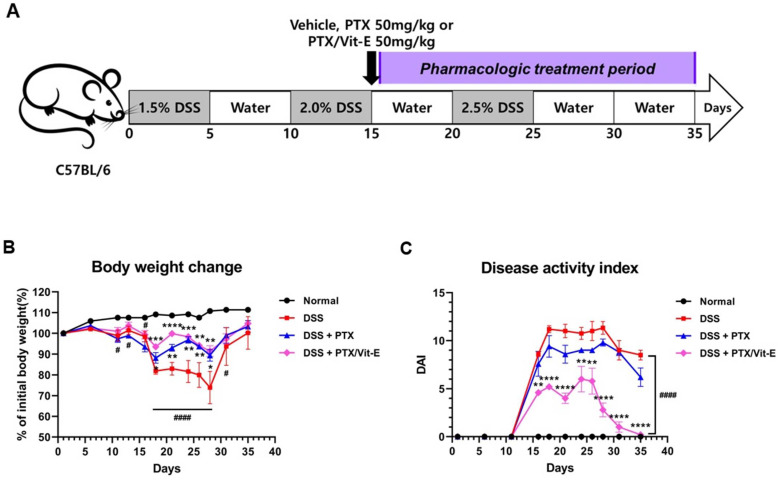

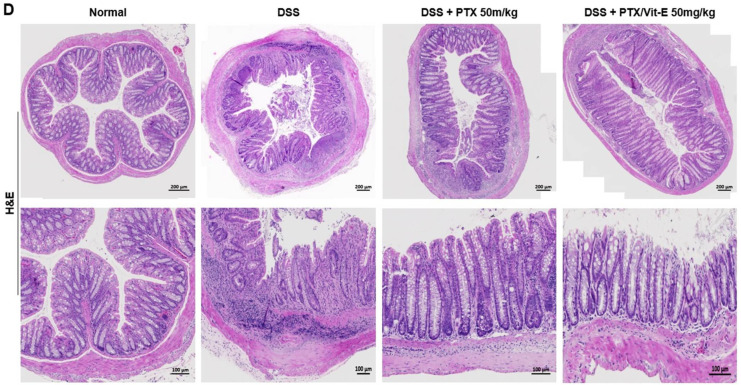
The co-administration with PTX and Vit-E improves disease activity in DSS-induced murine models of IBD. (**A**) Schematic diagram of the in vivo study design (n = 5 per group). The gray-colored regions indicate DSS treatment periods and the purple-colored region indicates the pharmacologic treatment period (e.g., vehicle, PTX, Vit-E). To induce colitis in mice, mice in all groups except the Normal group were administered 1.5–2.5% DSS in drinking water for three 5-day cycles. Afterwards, the DSS-induced colitis mice were i.p. injected with vehicle, PTX, or PTX/Vit-E, 3 times per week during the pharmacologic treatment period (from day 16 to day 35). (**B**) The percentage change (%) of initial body weight. (**C**) Disease activity index score; DAI = weight loss score + stool consistency score + hematochezia score (**B**,**C**) The data were presented as mean ± SEM and analyzed by one-way ANOVA, followed by Dunnett’s test. *p* < 0.05 was considered significant (^#^ *p* < 0.05; ^####^ *p* < 0.0001 versus the Normal group; * *p* < 0.05; ** *p* < 0.01; *** *p* < 0.001; **** *p* < 0.0001 versus the DSS-only-treated group). (**D**) H&E-stained colon sections, original magnification ×200, scale bar 200 μm; 100 μm. In comparison to normal morphology of crypts with absence of inflammation from colon sections in the Normal group, we observed severe inflammatory infiltration, predominantly in the lamina propria and submucosa, with degeneration of the mucosal epithelium, decreased goblet cells, loss of crypt architecture, and cryptitis/formation of crypt abscess from those in the DSS-only-treated group. The treatment with PTX and Vit-E seemed to restore colonic tissue damage.

Following our in vitro studies supporting the ameliorative role of the combination of PTX and Vit-E in intestinal fibrosis, we further assessed the beneficial effects of the combination therapy in IBD in vivo models using DSS-induced colitis. We randomly divided mice into four groups as follows: Normal (n = 5); DSS + vehicle (n = 5); DSS + 50 mg/kg PTX (n = 5); DSS + 50 mg/kg PTX + 50 mg/kg Vit-E (n = 5). A schematic overview of the animal study design is described in Figure 5A. Throughout the animal experiment, mice were monitored for clinical symptoms of IBD. The body weight of the mice that had drunk only water remained stable or increased for 35 days (Figure 5B). Meanwhile, noticeable weight loss from initial body weight was evident in the DSS group compared to those in the normal group. On day 21, the mice treated with only DSS had lost approximately 17% of their weight and, on day 28, roughly 26%. However, the mice in the DSS + PTX group and the DSS + PTX/Vit-E group experienced less weight loss than those in the DSS-only-treated group. On day 21, mice in the DSS + PTX group had lost approximately 7% of their original body weight and the body weight of the mice in the DSS + PTX/Vit-E group was almost similar to original body weight (99.87 ± 1.731% of initial body weight). On day 28, that of the mice in the DSS + PTX group decreased by 10.6% and that of the mice in the DSS + PTX/Vit-E group decreased by 8.6%. During the 26–35-day recovery period, the mice in the three experimental groups (DSS + vehicle, DSS + PTX, DSS + PTX/Vit-E) steadily gained weight. To further assess the severity of colitis, the combined average daily scores of stool consistency, stool bleeding and body weight loss were used to generate the DAI scores. A higher DAI score is associated with more severe colitis. DSS caused severe diarrhea, hematochezia, and sustained weight loss accompanied by general weakness, whereas the mice that were administered with 50 mg/kg of PTX and Vit-E developed the least severe colitis symptoms of the mice, which ingested DSS in drinking water. Consistently, while the DAI score increased after DSS intake, it was attenuated in the DSS + PTX group and markedly attenuated in the DSS + PTX/Vit-E group (Figure 5C). By day 18 and day 28, the DSS group exhibited the highest DAI score, 11. In the DSS+PTX/Vit-E group, this was 5 on day 18, and 3 on day 28.

Cross-sections of colonic tissue samples stained with H&E showed that there were numerous occasions of inflammatory infiltration, especially into the lamina propria and submucosa, outstanding destruction of crypt architecture, and cryptitis/formation of crypt abscess in the DSS group, compared to the intact structure of crypts in the Normal group (Figure 5D). The H&E-stained samples also showed that the co-administration with PTX and Vit-E alleviated the extensive inflammatory response and tissue injuries caused by DSS within the colon.

### 3.5. The Combination of PTX and Vit-E Ameliorates Intestinal Fibrosis in Animal Models of IBD

**Figure 6:**
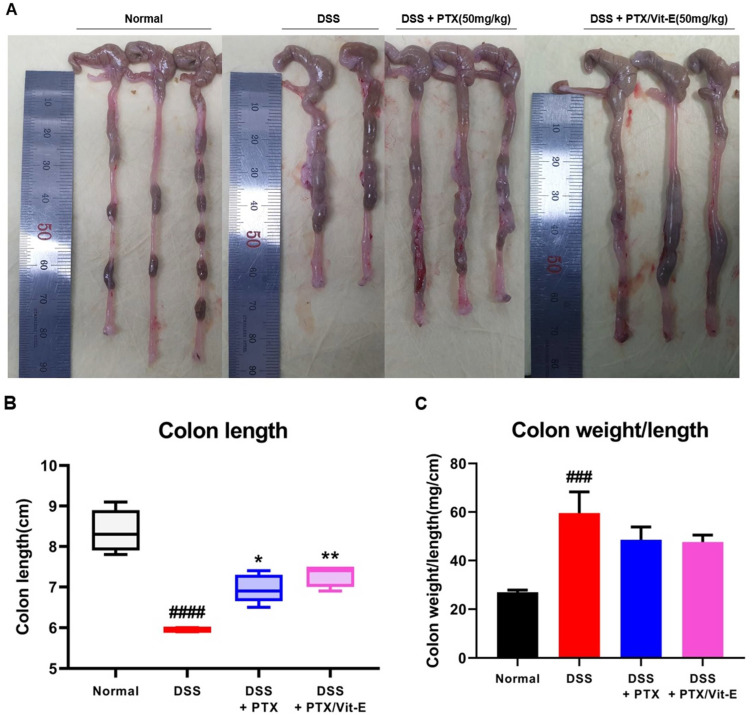

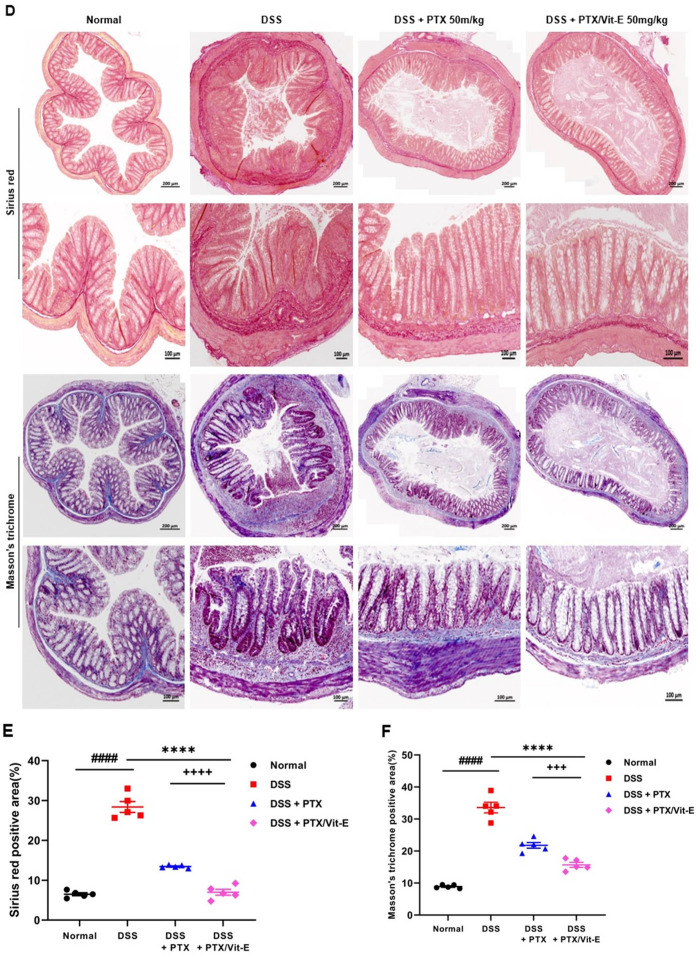

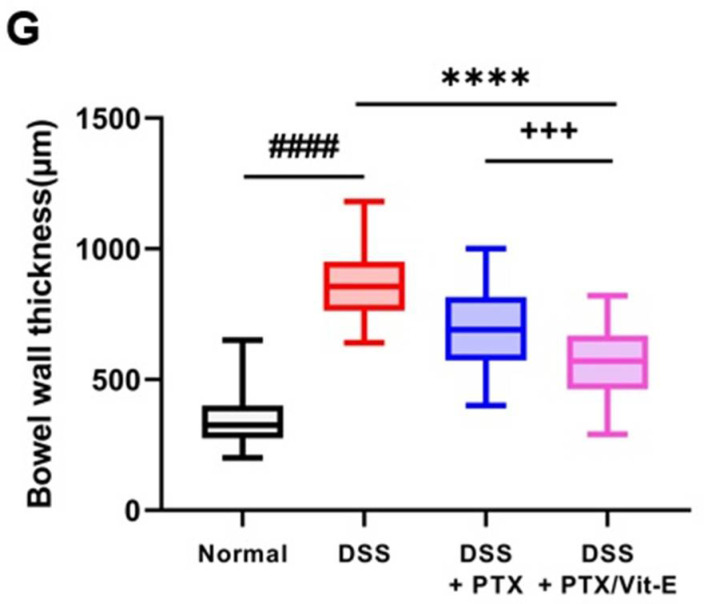
The combined PTX and Vit-E treatment prevents the progression of colonic fibrosis with recovery from thickening and shortening of colon in vivo. (**A**) Representative gross pictures of colons extracted from each group. The colon length was measured from the cecum to the anus. (**B**) The length of colon from the DSS-only group tended to definitely shorten compared with that from the Normal group. On the other hand, the combined PTX and Vit-E treatment restored the colon length slightly more than the single treatment with PTX. (**C**) The weight/length of colons from the DSS-only group tended to be heavier than that from the Normal group. However, the combined PTX and Vit-E treatment seemed to prevent the weight of colon from getting heavier. (**D**) Representative images of Sirius red and Masson’s trichrome staining of colon tissue in each group, original magnification ×200, scale bar 200 μm; 100 μm. The ‘red area’ stained with Sirius red and the ‘blue area’ stained with Masson’s trichrome indicate fibrotic area. Sirius red and Masson’s trichrome staining represented augmented collagen deposition, particularly in the submucosal layer, within the colons of the DSS-only-treated mice. In contrast, fibrosis-related collagen deposition was attenuated in the DSS + PTX/Vit-E group. (**E**,**F**) Quantification of colonic fibrosis, as assessed by Sirius red or Masson’s trichrome staining. The results revealed that exposure to DSS triggered the excessive accumulation of ECM components, leading to the progression of fibrosis, whereas co-administration with PTX and Vit-E alleviated DSS-induced-fibrosis to a greater extent than the single treatment with PTX. (**G**) The measurements of the thickness of histological bowel wall. The colon wall thickness in the DSS group was significantly greater than that in the Normal group. The combined PTX and Vit-E treatment hindered the colon wall from thickening in the DSS-induced colitis significantly more than the single treatment with PTX. The data were presented as mean ± SEM and analyzed by one-way ANOVA, followed by Dunnett’s test or unpaired two-tailed Student’s *t*-test. *p* < 0.05 was considered significant (^###^ *p* < 0.001; ^####^ *p* < 0.0001 versus the Normal group; * *p* < 0.05; ** *p* < 0.01; **** *p* < 0.0001 versus the DSS-only-treated group; ^+++^ *p* < 0.001; ^++++^ *p* < 0.0001, the DSS+PTX/Vit-E group versus the DSS+PTX group).

Compared with the colons from the Normal group, those deriving from the DSS group tended to shorten, be thicker, and weigh more (Figure 6). The mean colon length in the DSS group was 5.95 ± 0.05 cm, which was definitely shorter than 8.38 ± 0.2354 cm, which was found in the Normal group (*p* < 0.0001). The mean colon length in the DSS + PTX group was 6.96 ± 0.1568 cm, which was longer than that in the DSS group (*p* < 0.05). The mean colon length in the DSS + PTX/Vit-E group was 7.28 ± 0.12 cm, which was longer than that in the DSS + PTX group as well as that in the DSS group (*p* < 0.01) and was likely to be close to that in the Normal group. There was no statistical significance between the DSS + PTX group and DSS + PTX/Vit-E group. The mean colon weight/length in the DSS group was 59.59 ± 8.743, which obviously was heavier than 26.93 ± 0.8871, as in the Normal group (*p* < 0.001). The mean colon weight/length in the DSS + PTX/Vit-E group was 47.62 ± 2.914, which was lighter than that in the DSS group, but the results were not statistically significant. The mean colon wall thickness in the DSS group, 865 ± 19.74 μm was distinctly greater than 345.4 ± 14.38 μm, as in the Normal group (*p* < 0.0001). The mean colon wall thickness in the DSS + PTX/Vit-E group, 564.8 ± 22.95 μm was thinner than that in the DSS + PTX group, 680.8 ± 21.91 μm (*p* < 0.001) as well as that in the DSS group (*p* < 0.0001).

We stained colon segments with Sirius red and Masson’s trichrome to evaluate colonic fibrosis (Figure 6D). Sirius red and Masson’s trichrome staining represented obvious collagen deposition, particularly in the submucosal layer, in the DSS-only-treated mice. By contrast, fibrosis-related collagen deposition was alleviated in the DSS + PTX/Vit-E group. The fibrotic area was quantified from the ‘blue area’, stained with Masson’s trichrome (MT), or ‘red area’, stained with Sirius red (SR) (Figure 6E,F). The degree of colon tissue fibrosis was significantly reduced in the DSS + PTX/Vit-E group in comparison to the DSS group (MT: 15.68 ± 0.7829% vs. 33.59 ± 1.641%, *p* < 0.0001; SR: 6.979 ± 0.7444% vs. 28.40 ± 1.369%, *p* < 0.0001). Administration with PTX and Vit-E could appear to protect against ECM deposition, which contributed to the suppressed intestinal fibrosis in DSS-induced IBD murine models. Clearly, the efficacy of the combination of PTX and Vit-E was better than that of PTX alone (MT: 15.68 ± 0.7829% vs. 21.78 ± 0.8869%, *p* < 0.001; SR: 6.979 ± 0.7444% vs. 13.45 ± 0.1644%, *p* < 0.0001).

## 4. Discussion

As the prevalence of IBD has steadily increased internationally, the importance of a long-term treatment plan to delay the progression of the incurable disease has been emphasized. As IBD is understood as a complex and chronic multifactorial disease, a multidisciplinary therapeutic strategy has recently been highlighted and new approaches or agents have been developed. Historically, the mainstay of UC therapy has been 5-aminosalicylic acid (5-ASA), while short-term steroid therapy is effective for severe flares and escalation to immunomodulators or biologics such as tumor necrosis factor-α (TNF-α) inhibitors needs to be considered after 5-ASA failure. On the other hand, corticosteroids have most commonly been used to induce remission in CD. Likewise, as with UC, immunomodulators or biologics are recommended for alternatives after first-line therapy failure. Immunomodulators and biologics may be used, either in combination or as a monotherapy. Recently, newly approved and upcoming treatment options include TNF-α inhibitors, S1P-receptor modulators, IL-12/IL-23 inhibitors, JAK/STAT inhibitors, stem-cell transplant and fecal microbiota transplant (FMT), etc. Although the treatment goal for IBD has been mainly focused on controlling inflammation, we also pursue the clinical development of a novel anti-fibrotic agent because intestinal fibrosis, which is a common complication in patients with long-term IBD, is not ameliorated by alleviating inflammation.

TGF-β1, one of the most potent profibrotic factors, plays a crucial role in the pathogenesis of intestinal fibrosis, including the proliferation of fibroblasts, differentiation into myofibroblasts, and excessive production of ECM [58]. The activation of TGF-β1 signaling in the colonic epithelium promotes colonic inflammation, accompanied by thickening of the smooth muscle tissues and the localized deposition of ECM within the bowel wall, eventually leading to fibrotic process [17]. In the 2010 Flier et al. study, TGF-β1-driven epithelial-mesenchymal transition (EMT) contributed to intestinal fibrosis in a rodent model of CD, and the inhibition of TGF-β1 signaling prevented this process, as well as fibrosis [59]. Targeting TGF-β1 signaling pathways is key to developing a novel anti-fibrotic agent for IBD patients. TGF-β1-stimulated HIMFs are regarded as a good cellular model to evaluate the efficacy of an anti-fibrotic agent as a novel therapy to IBD.

Understanding the role of intestinal myofibroblasts is also essential to identifying the pathophysiology of intestinal fibrosis. Once myofibroblasts are activated by paracrine proinflammatory cytokines and chemokines in the microenvironment, the cells facilitate wound-healing process, most of which is primarily mediated by TGF-β1. Activated myofibroblasts not only migrate toward the lesion center, but also secrete ECM components, including collagen, TGF-β1, CTGF, fibronectin, IGF-I/II, platelet-derived growth factor and basic fibroblast growth factor, as well as various cytokines and chemokines, resulting in a thickening of the mesenchymal cell layer. If dysregulated, myofibroblasts produce excess ECM components, converting type IV collagen into collagen types I and III, which are gradually deposited into fibrillar ECM and distort normal tissue architecture, with an increase in tissue stiffness and progressive fibrosis, leading to tissue remodeling [60]. The expression of CTGF, a critical downstream mediator for TGF-β1-induced ECM production, is enhanced by many cytokines (e.g., TGF-β, VEGF, integrins) and conditions associated with pathophysiology in fibrotic tissue [61]. CTGF prompts myofibroblast formation, and increases the expression of different cytokines, which, in turn, further stimulate the expression of CTGF, generating positive feedback loops [62,63]. The TGF-β-triggered fibrogenic signaling on intestinal myofibroblasts is propagated through both Smad-dependent and Smad-independent pathways [7,64,65]. When Smad-dependent TGF-β1 signaling begins, the active homodimer form of TGF-β1 binds to the TGF-β receptor2 (TGFR2), which then recruits and activates TGF-β receptor1 (TGFR1). The active TGFR1 then phosphorylates Smad2 and Smad3, which form a complex with Smad4 and translocate to the nucleus. The Smad3 component of the complex directly binds to gene promoters to induce the transcription of profibrotic molecules, including α-SMA, collagen I, fibronectin, and tissue inhibitors of metalloproteinases (TIMPs), which facilitate myofibroblast activation and ECM deposition. On the other hand, TGF-β1 can also activate MAPKs (e.g., p38, JNK, ERK) in a Smad-independent manner. MAPKs can phosphorylate the linker region of Smad proteins to modulate Smad3 transcriptional activity and enhance gene transcription of profibrotic molecules [26]. As found in previous studies, we verified that TGF-β1 enhances the expression of ECM components and α-SMA through the promotion of both Smad and non-Smad signalings in HIMFs [21,22,27,66,67,68].

PTX, a methylxanthine derivative, is a non-selective PDE inhibitor, which raises intracellular adenosine 3′,5′-cyclic monophosphate (cAMP), activates protein kinase A (PKA), inhibits TNF and leukotriene synthesis, and has been reported to reduce inflammation and innate immunity. Cyclic nucleotide PDEs are the cellular enzymes responsible for the hydrolysis of phosphodiester bonds in two second messengers; cAMP or guanosine 3′,5′-cyclic monophosphate (cGMP). The elevated levels of cAMP or cGMP were demonstrated to exhibit anti-inflammatory and anti-fibrotic properties [69,70,71]. In a similar vein, PDE inhibitors also have been proven to induce anti-inflammatory and anti-fibrotic effects in different cellular or animal models. Even though the exact mechanism of these inhibitory effects on inflammation and fibrosis in various organs was not unveiled, these effects are elicited through modulation of the cAMP/PKA/CREB pathway, cGMP signaling and TGF-β/Smad-dependent signaling [72,73,74]. In this study, PTX not only decreased TGF-β1-induced-production of collagen and the expression of profibrotic molecules on HIMFs, but it also alleviated colonic fibrosis in DSS-induced IBD murine models. On the other hand, we found that PTX suppressed the phosphorylation of JNK and ERK, but had no meaningful effect on the phosphorylation of Smad2 in TGF-β1-stimulated HIMFs. Similarly in other studies, it did not interfere with TGF-β1-driven activation of Smad2 [41,75]. Thus, the underlying mechanism responsible for its anti-fibrotic effects may be illustrated by the interruption of Smad-independent TGF-β pathways via cAMP/PKA signaling.

Vit-E is a strong antioxidant, which protects the phospholipid bilayer of the plasma membranes against free radical oxidation. It has also been proven to attenuate liver fibrosis, renal fibrosis, and radiation-induced fibrosis [47,48,49,50,51,76]. The combination of PTX and Vit-E had a potent anti-fibrotic effect in prior studies and its anti-fibrotic effect was mainly thought to be involved in blocking TGF-β1 signaling [39,52,53,54]. In this study, we demonstrated that the combination treatment with PTX and Vit-E reduced the expression of fibrogenic molecules more than either treatment, alone, in vitro in HIMFs, and mitigated the degree of colonic fibrosis more than monotherapy with PTX in an in vivo IBD model. Moreover, the present in vivo results indicate that combined PTX and Vit-E treatment may prevent body weight loss and improve DAI, which reflects the severity of IBD. We could see the therapeutic potential of the combination of PTX and Vit-E for IBD. According to a Western blot analysis exploring the mechanisms of the anti-fibrotic effects of PTX and Vit-E, Vit-E pretreatment inhibited the phosphorylation of JNK, ERK and Smad2, whereas PTX pretreatment subdued the phosphorylation of JNK and ERK, but not Smad2 phosphorylation. Taken together, the anti-fibrotic properties of PTX could be explained by the non-Smad TGF-β pathways and Vit-E properties are associated with TGF-β/Smad and non-Smad pathways, suggesting that a greater anti-fibrotic effect of the combination of PTX and Vit-E is attributed to the blockade of both Smad and non-Smad pathways.

In the present animal experiment, we discovered that the colons from the DSS group tended to clearly shorten and thicken compared to those from the Normal group. Intestinal stricture in CD, developing as a result of fibrosis progression, which mainly occurs in the submucosa, is manifested by a thickening of the intestinal wall due to the hyperplasia of smooth muscle cells [77]. Likewise, the muscularis mucosae in the area of strictured intestine in UC was proven to be markedly thickened and linked with progressive fibrosis [78]. The accumulation of ECM and thickening of the muscularis mucosae in the colon of UC patients with both short- and long-standing disease resulted in a shortening and increased rigidity of the colon. Eventually, end-stage UC may be characterized by a ‘lead-pipe colon’, with a shortened, stiff, and narrowed colon with loss of haustrations [79,80]. Accordingly, in our in vivo study, shortening and thickening of the colon with the augmented deposition of ECM in the DSS group substantiate the idea that DSS-induced colitis murine models can safely be regarded as established animal models of IBD. The findings also showed that administration of the combination of PTX and Vit-E hindered DSS-induced shortening and thickening of the colon, suggesting the combination therapy may improve intestine flexibility.

There are some limitations to this study. The cellular study was performed on HIMFs from healthy tissue, not on fibroblasts from diseased tissue, so the results can partially be translated to diseased cells. The murine models, although significant, cannot fully resemble IBD behaviors in humans or clearly be divided into CD or UC models. Thus, further studies in humans are needed to evaluate the efficacy, safety, and possible dosing (effective and not harmful) of PTX and Vit-E in vivo. Finally, accurate criteria for determination of the degree of intestinal fibrosis in vivo have not actually been established to date. Fibrosis biomarkers, as well as diagnostic tools that can be used to identify and quantify intestinal fibrosis in vivo, should be developed and validated.

In summary, the data have shown that the combination of PTX and Vit-E exerted significant anti-fibrotic effects in both HIMFs and in vivo IBD models. These anti-fibrotic effects could be thought to be involved in the disruption of the Ras/MEK/ERK, JNK, and Smad signaling pathways. Our research may offer new hope to IBD patients, presenting a new approach for IBD therapy, the development of an anti-fibrotic agent. We anticipate that long-term combination therapy with PTX and Vit-E would slow the progression of intestinal fibrosis and preserve gut resilience, leading to improvements in quality of life. Furthermore, these findings imply that Vit-E supplementation can help to maintain the flexibility of the intestine in IBD patients, reminding us of the importance of vitamin supplementation. For future research, we expect to conduct clinical trials for the beneficial effects of the combination therapy with PTX and Vit-E on IBD patients.

## Data Availability

The data presented in this study are available on reasonable request from the corresponding author.

## Abbreviations

IBD: Inflammatory bowel disease, CD: Crohn’s disease, UC: Ulcerative colitis, FAP: Fibroblast activation protein, NETs: Neutrophil extracellular traps, TGF-β1: Transforming growth factor-β1, α-SMA: α-smooth muscle actin, CTGF: Connective tissue growth factor, ERK: Extracellular signal-regulated kinase, JNK: c-Jun N-terminal kinase, MAPK: Mitogen-activated protein kinase, PTX: Pentoxifylline, TNBS: 2,4,6-trinitrobenzene sulfonic acid, PDE: Phosphodiesterase, FDA: Food and Drug Administration, GAG: Glycosaminoglycan, Vit-E: Vitamin-E, MMP-1: Matrix metalloproteinase-1, HIMFs: Human primary intestinal myofibroblasts, DSS: Dextran sulfate sodium, DAI: Disease activity index, ECM: Extracellular matrix, MT: Masson’s trichrome, SR: Sirius red, TNF: Tumor necrosis factor, S1P-receptor: Sphingosine-1-phosphate receptor, EMT: Epithelial-mesenchymal transition, IGF-I/II: Insulin-like growth factor-I/II, VEGF: Vascular endothelial growth factor, TGFR2: TGF-β receptor2, TGFR1: TGF-β receptor1, TIMPs: Tissue inhibitors of metalloproteinases, cAMP: Adenosine 3′,5′-cyclic monophosphate, PKA: Protein kinase A, cGMP: Guanosine 3′,5′-cyclic monophosphate, CREB: cAMP response element-binding protein

## References

[1] Latella G., Sferra R., Speca S., Vetuschi A., Gaudio E. (2013). Can we prevent, reduce or reverse intestinal fibrosis in IBD?. Eur. Rev. Med. Pharmacol. Sci..

[2] McDowell C., Farooq U., Haseeb M. (2022). Inflammatory Bowel Disease.

[3] Burke J.P., Mulsow J.J., O’keane C., Docherty N.G., Watson R.W., O’connell P.R. (2007). Fibrogenesis in Crohn’s disease. Am. J. Gastroenterol..

[4] Rieder F., Fiocchi C. (2008). Intestinal fibrosis in inflammatory bowel disease–Current knowledge and future perspectives. J. Crohn’s Colitis.

[5] Rieder F., Fiocchi C. (2009). Intestinal fibrosis in IBD-a dynamic, multifactorial process. Nat. Rev. Gastroenterol. Hepatol..

[6] Thia K.T., Sandborn W.J., Harmsen W.S., Zinsmeister A.R., Loftus E.V. (2010). Risk factors associated with progression to intestinal complications of Crohn’s disease in a population-based cohort. Gastroenterology.

[7] Lawrance I.C., Rogler G., Bamias G., Breynaert C., Florholmen J., Pellino G., Reif S., Speca S., Latella G. (2017). Cellular and molecular mediators of intestinal fibrosis. J. Crohn’s Colitis.

[8] Longo W.E., Virgo K.S., Bahadursingh A.N., Johnson F.E. (2003). Patterns of disease and surgical treatment among United States veterans more than 50 years of age with ulcerative colitis. Am. J. Surg..

[9] Van Assche G., Geboes K., Rutgeerts P. (2004). Medical therapy for Crohn’s disease strictures. Inflamm. Bowel Dis..

[10] Rieder F., Bettenworth D., Imai J., Inagaki Y. (2016). Intestinal fibrosis and liver fibrosis: Consequences of chronic inflammation or independent pathophysiology?. Inflamm. Intest. Dis..

[11] Vetuschi A., Pompili S., Gaudio E., Latella G., Sferra R. (2018). PPAR-γ with its anti-inflammatory and anti-fibrotic action could be an effective therapeutic target in IBD. Eur. Rev. Med. Pharmacol. Sci..

[12] Johnson L.A., Luke A., Sauder K., Moons D.S., Horowitz J.C., Higgins P.D. (2012). Intestinal fibrosis is reduced by early elimination of inflammation in a mouse model of IBD: Impact of a “top-down” approach to intestinal fibrosis in mice. Inflamm. Bowel Dis..

[13] Santacroce G., Lenti M.V., Di Sabatino A. (2022). Therapeutic Targeting of Intestinal Fibrosis in Crohn’s Disease. Cells.

[14] Border W.A., Noble N.A. (1994). Transforming growth factor β in tissue fibrosis. N. Engl. J. Med..

[15] Hinz B., Gabbiani G. (2003). Cell-matrix and cell-cell contacts of myofibroblasts: Role in connective tissue remodeling. Thromb. Haemost..

[16] Desmoulière A., Geinoz A., Gabbiani F., Gabbiani G. (1993). Transforming growth factor-beta 1 induces alpha-smooth muscle actin expression in granulation tissue myofibroblasts and in quiescent and growing cultured fibroblasts. J. Cell Biol..

[17] Vallance B.A., Gunawan M.I., Hewlett B., Bercik P., Van Kampen C., Galeazzi F., Sime P.J., Gauldie J., Collins S.M. (2005). TGF-β1 gene transfer to the mouse colon leads to intestinal fibrosis. Am. J. Physiol.-Gastrointest. Liver Physiol..

[18] Yun S.M., Kim S.H., Kim E.H. (2019). The Molecular Mechanism of Transforming Growth Factor-β Signaling for Intestinal Fibrosis: A Mini-Review. Front. Pharmacol..

[19] Babyatsky M.W., Rossiter G., Podolsky D.K. (1996). Expression of transforming growth factors alpha and beta in colonic mucosa in inflammatory bowel disease. Gastroenterology.

[20] Rosenbloom J., Castro S.V., Jimenez S.A. (2010). Narrative review: Fibrotic diseases: Cellular and molecular mechanisms and novel therapies. Ann. Intern. Med..

[21] Johnson L.A., Rodansky E.S., Haak A.J., Larsen S.D., Neubig R.R., Higgins P.D. (2014). Novel Rho/MRTF/SRF inhibitors block matrix-stiffness and TGF-beta-induced fibrogenesis in human colonic myofibroblasts. Inflamm. Bowel Dis..

[22] Biancheri P., Giuffrida P., Docena G.H., MacDonald T.T., Corazza G.R., Di Sabatino A. (2014). The role of transforming growth factor(TGF)-beta in modulating the immune response and fibrogenesis in the gut. Cytokine Growth Factor Rev..

[23] Derynck R., Zhang Y.E. (2003). Smad-dependent and Smad-independent pathways in TGF-beta family signalling. Nature.

[24] Medina C., Santos-Martinez M.J., Santana A., Paz-Cabrera M.C., Johnston M.J., Mourelle M., Salas A., Guarner F. (2011). Transforming growth factor-beta type 1 receptor (ALK5) and Smad proteins mediate TIMP-1 and collagen synthesis in experimental intestinal fibrosis. J. Pathol..

[25] Massagué J., Wotton D. (2000). Transcriptional control by the TGF-beta/Smad signaling system. EMBO J..

[26] Meng X.M., Nikolic-Paterson D.J., Lan H.Y. (2016). TGF-β: The master regulator of fibrosis. Nat. Rev. Nephrol..

[27] Mulsow J.J., Watson R.W., Fitzpatrick J.M., O’Connell P.R. (2005). Transforming growth factor-beta promotes pro-fibrotic behavior by serosal fibroblasts via PKC and ERK1/2 mitogen activated protein kinase cell signaling. Ann. Surg..

[28] Kulkarni A.A., Thatcher T.H., Olsen K.C., Maggirwar S.B., Phipps R.P., Sime P.J. (2011). PPAR-γ ligands repress TGF-β-induced myofibroblast differentiation by targeting the PI3K/Akt pathway: Implications for therapy of fibrosis. PLoS ONE.

[29] Mu Y., Gudey S.K., Landström M. (2012). Non-Smad signaling pathways. Cell Tissue Res..

[30] Wengrower D., Zanninelli G. (2004). Prevention of fibrosis in experimental colitis by captopril: The role of tgf-beta1. Inflamm. Bowel Dis..

[31] Holvoet T., Devriese S., Castermans K., Boland S., Leysen D., Vandewynckel Y.P., Devisscher L., Van den Bossche L., Van Welden S., Dullaers M. (2017). Treatment of Intestinal Fibrosis in Experimental Inflammatory Bowel Disease by the Pleiotropic Actions of a Local Rho Kinase Inhibitor. Gastroenterology.

[32] Li C., Flynn R.S. (2013). Increased activation of latent TGF-β1 by αVβ3 in human Crohn’s disease and fibrosis in TNBS colitis can be prevented by cilengitide. Inflamm. Bowel Dis..

[33] Sun Y., Zhang Y., Chi P. (2018). Pirfenidone suppresses TGF-β1-induced human intestinal fibroblasts activities by regulating proliferation and apoptosis via the inhibition of the Smad and PI3K/AKT signaling pathway. Mol. Med. Rep..

[34] Li G., Ren J., Hu Q., Deng Y., Chen G., Guo K., Li R., Li Y., Wu L., Wang G. (2016). Oral pirfenidone protects against fibrosis by inhibiting fibroblast proliferation and TGF-β signaling in a murine colitis model. Biochem. Pharmacol..

[35] Peterson T.C., Peterson M.R., Raoul J.L. (2011). The effect of pentoxifylline and its metabolite-1 on inflammation and fibrosis in the TNBS model of colitis. Eur. J. Pharmacol..

[36] Karatay E., Gül Utku Ö., Erdal H., Arhan M., Önal İ.K., Ibiş M., Ekinci Ö., Yilmaz Demirtaş C., G Dumlu Ş. (2017). Pentoxifylline attenuates mucosal damage in an experimental model of rat colitis by modulating tissue biomarkers of inflammation, oxidative stress, and fibrosis. Turk. J. Med. Sci..

[37] Okunieff P., Augustine E., Hicks J.E., Cornelison T.L., Altemus R.M., Naydich B.G., Ding I., Huser A.K., Abraham E.H., Smith J.J. (2004). Pentoxifylline in the treatment of radiation-induced fibrosis. J. Clin. Oncol..

[38] Hille A., Christiansen H., Pradier O., Hermann R.M., Siekmeyer B., Weiss E., Hilgers R., Hess C.F., Schmidberger H. (2005). Effect of pentoxifylline and tocopherol on radiation proctitis/enteritis. Strahlenther. Onkol..

[39] Hamama S., Gilbert-Sirieix M., Vozenin M.C., Delanian S. (2012). Radiation-induced enteropathy: Molecular basis of pentoxifylline-vitamin E anti-fibrotic effect involved TGF-β1 cascade inhibition. Radiother. Oncol..

[40] Satapathy S.K., Sakhuja P., Malhotra V., Sharma B.C., Sarin S.K. (2007). Beneficial effects of pentoxifylline on hepatic steatosis, fibrosis and necroinflammation in patients with non-alcoholic steatohepatitis. J. Gastroenterol. Hepatol..

[41] Lin S.L., Chen R.H., Chen Y.M., Chiang W.C., Lai C.F., Wu K.D., Tsai T.J. (2005). Pentoxifylline attenuates tubulointerstitial fibrosis by blocking Smad3/4-activated transcription and profibrogenic effects of connective tissue growth factor. J. Am. Soc. Nephrol..

[42] Hassan I., Dorjay K., Anwar P. (2014). Pentoxifylline and its applications in dermatology. Indian Dermatol. Online J..

[43] Berman B., Duncan M.R. (1989). Pentoxifylline inhibits normal human dermal fibroblast in vitro proliferation, collagen, glycosaminoglycan, and fibronectin production, and increases collagenase activity. J. Investig. Dermatol..

[44] Ricciarelli R., Maroni P., Ozer N., Zingg J.M., Azzi A. (1999). Age-dependent increase of collagenase expression can be reduced by alpha-tocopherol via protein kinase C inhibition. Free Radic. Biol. Med..

[45] Akeson A.L., Woods C.W., Mosher L.B., Thomas C.E., Jackson R.L. (1991). Inhibition of IL-1 beta expression in THP-1 cells by probucol and tocopherol. Atherosclerosis.

[46] Chojkier M., Houglum K., Lee K.S., Buck M. (1998). Long- and short-term D-alpha-tocopherol supplementation inhibits liver collagen alpha1(I) gene expression. Am. J. Physiol..

[47] Soylu A.R., Aydogdu N., Basaran U.N., Altaner S., Tarcin O., Gedik N., Umit H., Tezel A., Dokmeci G., Baloglu H. (2006). Antioxidants vitamin E and C attenuate hepatic fibrosis in biliary-obstructed rats. World J. Gastroenterol..

[48] Zamin I., Mattos A.A., Mattos A.Z., Coral G., Santos D., Rhoden C. (2010). The vitamin E reduces liver lipoperoxidation and fibrosis in a model of nonalcoholic steatohepatitis. Arq. Gastroenterol..

[49] Bese N.S., Munzuroglu F., Uslu B., Arbak S., Yesiladali G., Sut N., Altug T., Ober A. (2007). Vitamin E protects against the development of radiation-induced pulmonary fibrosis in rats. Clin. Oncol..

[50] Tasanarong A., Kongkham S., Duangchana S., Thitiarchakul S., Eiam-Ong S. (2011). Vitamin E ameliorates renal fibrosis by inhibition of TGF-beta/Smad2/3 signaling pathway in UUO mice. J. Med. Assoc. Thai..

[51] Tasanarong A., Kongkham S., Thitiarchakul S., Eiam-Ong S. (2011). Vitamin E ameliorates renal fibrosis in ureteral obstruction: Role of maintaining BMP-7 during epithelial-to-mesenchymal transition. J. Med. Assoc. Thai..

[52] Kaya V., Yazkan R., Yıldırım M., Doguc D.K., Süren D., Bozkurt K.K., Yuksel O., Demirpence O., Sen C.A., Yalçın A.Y. (2014). The relation of radiation-induced pulmonary fibrosis with stress and the efficiency of antioxidant treatment: An experimental study. Med. Sci. Monit..

[53] Chiao T.B., Lee A.J. (2005). Role of Pentoxifylline and Vitamin E in Attenuation of Radiation-Induced Fibrosis. Ann. Pharmacother..

[54] Jacobson G., Bhatia S., Smith B.J., Button A.M., Bodeker K., Buatti J. (2013). Randomized trial of pentoxifylline and vitamin E vs standard follow-up after breast irradiation to prevent breast fibrosis, evaluated by tissue compliance meter. Int. J. Radiat. Oncol. Biol. Phys..

[55] Liu H., Xiong M., Xia Y.F., Cui N.J., Lu R.B., Deng L., Lin Y.H., Rong T.H. (2009). Studies on pentoxifylline and tocopherol combination for radiation-induced heart disease in rats. Int. J. Radiat. Oncol. Biol. Phys..

[56] Ng Y.Y., Chen Y.M., Tsai T.J., Lan X.R., Yang W.C., Lan H.Y. (2009). Pentoxifylline inhibits transforming growth factor-beta signaling and renal fibrosis in experimental crescentic glomerulonephritis in rats. Am. J. Nephrol..

[57] Hung K.Y., Huang J.W., Chiang C.K., Tsai T.J. (2008). Preservation of peritoneal morphology and function by pentoxifylline in a rat model of peritoneal dialysis: Molecular studies. Nephrol. Dial. Transplant..

[58] Speca S., Giusti I., Rieder F., Latella G. (2012). Cellular and molecular mechanisms of intestinal fibrosis. World J. Gastroenterol..

[59] Flier S.N., Tanjore H., Kokkotou E.G., Sugimoto H., Zeisberg M., Kalluri R. (2010). Identification of epithelial to mesenchymal transition as a novel source of fibroblasts in intestinal fibrosis. J. Biol. Chem..

[60] Li C., Kuemmerle J.F. (2020). The fate of myofibroblasts during the development of fibrosis in Crohn’s disease. J. Dig. Dis..

[61] Oliver N., Sternlicht M., Gerritsen K., Goldschmeding R. (2010). Could aging human skin use a connective tissue growth factor boost to increase collagen content?. J. Investig. Dermatol..

[62] Lipson K.E., Wong C., Teng Y., Spong S. (2012). CTGF is a central mediator of tissue remodeling and fibrosis and its inhibition can reverse the process of fibrosis. Fibrogenesis Tissue Repair.

[63] Yang H., Huang Y., Chen X., Liu J., Lu Y., Bu L., Xia L., Xiao W., Chen M., Nie Q. (2010). The role of CTGF in the diabetic rat retina and its relationship with VEGF and TGF-beta(2), elucidated by treatment with CTGFsiRNA. Acta Ophthalmol..

[64] Yang B., Zhang G., Elias M., Zhu Y., Wang J. (2020). The role of cytokine and immune responses in intestinal fibrosis. J. Dig. Dis..

[65] Biernacka A., Dobaczewski M., Frangogiannis N.G. (2011). TGF-β signaling in fibrosis. Growth Factors..

[66] Chen Q., Yang W., Wang X., Li X., Qi S., Zhang Y., Gao M.Q. (2017). TGF-β1 Induces EMT in Bovine Mammary Epithelial Cells Through the TGFβ1/Smad Signaling Pathway. Cell. Physiol. Biochem..

[67] Kaimori A., Potter J., Kaimori J., Wang C., Mezey E., Koteish A. (2007). Transforming Growth Factor-β1 Induces an Epithelial-to-Mesenchymal Transition State in Mouse Hepatocytes in vitro. J. Biol. Chem..

[68] Kang H.R., Cho S.J., Lee C.G., Homer R.J., Elias J.A. (2007). Transforming growth factor (TGF)-beta1 stimulates pulmonary fibrosis and inflammation via a Bax-dependent, bid-activated pathway that involves matrix metalloproteinase-12. J. Biol. Chem..

[69] Shen K., Johnson D.W., Gobe G.C. (2016). The role of cGMP and its signaling pathways in kidney disease. Am. J. Physiol.-Renal Physiol..

[70] Liu Y., Xu H., Geng Y., Xu D., Zhang L., Yang Y., Wei Z., Zhang B., Li S., Gao X. (2017). Dibutyryl-cAMP attenuates pulmonary fibrosis by blocking myofibroblast differentiation via PKA/CREB/CBP signaling in rats with silicosis. Respir. Res..

[71] Flores-Costa R., Duran-Güell M., Casulleras M., López-Vicario C., Alcaraz-Quiles J., Diaz A., Lozano J.J., Titos E., Hall K., Sarno R. (2020). Stimulation of soluble guanylate cyclase exerts antiinflammatory actions in the liver through a VASP/NF-κB/NLRP3 inflammasome circuit. Proc. Natl. Acad. Sci. USA.

[72] Wójcik-Pszczoła K., Chłoń-Rzepa G., Jankowska A., Ślusarczyk M., Ferdek P.E., Kusiak A.A., Świerczek A., Pociecha K., Koczurkiewicz-Adamczyk P., Wyska E. (2020). A Novel, Pan-PDE Inhibitor Exerts Anti-Fibrotic Effects in Human Lung Fibroblasts via Inhibition of TGF-β Signaling and Activation of cAMP/PKA Signaling. Int. J. Mol. Sci..

[73] Fehrholz M., Speer C.P., Kunzmann S. (2014). Caffeine and rolipram affect Smad signalling and TGF-β1 stimulated CTGF and transgelin expression in lung epithelial cells. PLoS ONE.

[74] Yougbare I. (2021). Alterations of cAMP/cGMP Signaling Pathways in Lupus Nephritis. J. Nephrol. Sci..

[75] Hung K.Y., Huang J.W., Chen C.T., Lee P.H., Tsai T.J. (2003). Pentoxifylline modulates intracellular signalling of TGF-beta in cultured human peritoneal mesothelial cells: Implications for prevention of encapsulating peritoneal sclerosis. Nephrol. Dial. Transplant..

[76] Shams S., Khan S., Ayaz M., Khan H.A., Hassan H. (2018). Effect of stem cell and vitamin E for the reduction of liver fibrosis. J. Appl. Environ. Biol. Sci..

[77] Honzawa Y., Yamamoto S., Okabe M., Seno H., Nakase H. (2021). Current Topics of the Mechanism of Intestinal Fibrosis in Crohn’s Disease. Immuno.

[78] Gordon I.O., Agrawal N., Willis E., Goldblum J.R., Lopez R., Allende D., Liu X., Patil D.Y., Yerian L., El-Khider F. (2018). Fibrosis in ulcerative colitis is directly linked to severity and chronicity of mucosal inflammation. Aliment. Pharmacol. Ther..

[79] de Bruyn J.R., Meijer S.L., Wildenberg M.E., Bemelman W.A., van den Brink G.R., D’Haens G.R. (2015). Development of fibrosis in acute and longstanding ulcerative colitis. J. Crohn’s Colitis.

[80] Giuffrida P., Pinzani M., Corazza G.R., Di Sabatino A. (2016). Biomarkers of intestinal fibrosis—One step towards clinical trials for stricturing inflammatory bowel disease. United Eur. Gastroenterol. J..

